# Giant panda foraging and movement patterns in response to bamboo shoot growth

**DOI:** 10.1007/s11356-017-0919-9

**Published:** 2018-01-10

**Authors:** Mingchun Zhang, Zhizhong Zhang, Zhong Li, Mingsheng Hong, Xiaoping Zhou, Shiqiang Zhou, Jindong Zhang, Vanessa Hull, Jinyan Huang, Hemin Zhang

**Affiliations:** 1 China Conservation and Research Center for the Giant Panda, Dujiangyan, Sichuan 611870 China; 2 Key Laboratory of Southwest China Wildlife Resources Conservation, China West Normal University, Ministry of Education, Nanchong, Sichuan 637009 China; 3 Genetic Engineering Research Center, School of Life Sciences, Chongqing University, Chongqing, 400044 China; 4 Department of Wildlife Ecology and Conservation, University of Florida, Gainesville, FL 32611 USA

**Keywords:** Giant panda (*Ailuropoda melanoleuca*), *Fargesia robusta*, Foraging patterns, Spatiotemporal variation

## Abstract

Diet plays a pivotal role in dictating behavioral patterns of herbivorous animals, particularly specialist species. The giant panda (*Ailuropoda melanoleuca*) is well-known as a bamboo specialist. In the present study, the response of giant pandas to spatiotemporal variation of bamboo shoots was explored using field surveys and GPS collar tracking. Results show the dynamics in panda-bamboo space-time relationships that have not been previously articulated. For instance, we found a higher bamboo stump height of foraged bamboo with increasing elevation, places where pandas foraged later in spring when bamboo shoots become more fibrous and woody. The time required for shoots to reach optimum height for foraging was significantly delayed as elevation increased, a pattern which corresponded with panda elevational migration patterns beginning from the lower elevational end of *Fargesia robusta* distribution and gradually shifting upward until the end of the shooting season. These results indicate that giant pandas can respond to spatiotemporal variation of bamboo resources, such as available shoots. Anthropogenic interference of low-elevation *F. robusta* habitat should be mitigated, and conservation attention and increased monitoring should be given to *F. robusta* areas at the low- and mid-elevation ranges, particularly in the spring shooting season.

## Introduction

Diet plays a pivotal role in shaping the behavior and population dynamics of herbivorous foragers, including shaping the niches they occupy in dynamic environments (Simpson and Raubenheimer 2012). Diet also informs the investigation of ecological flexibility, such as in predicting a species’ vulnerability to ecological perturbations (Hong et al. 2016). While generalist species may exhibit flexibility by having a greater diet breadth and broader geographical range, specialist species are often more vulnerable because they may have a limited diet and spatial distribution that may increase their risk of extinction (Clavel et al. 2011; Slayter et al. 2013; Ducatez et al. 2015). Foraging strategies of such specialist species are a central focus of study in animal ecology due to their influence on habitat selection, home range, social interactions, reproduction, and population regulation (Goss-Custard et al. 1995; Owen-Smith et al. 2010).

As bamboo specialists, giant pandas (*Ailuropoda melanoleuca*) are currently limited to around 25,000 km^2^ of suitable habitat in southwestern China. The estimated 1864 remaining giant pandas (State Forestry Administration of the People’s Republic of China 2015) are facing many human-induced threats including road construction, timber harvesting, and livestock grazing (Hull et al. 2014; Hong et al. 2015, 2016; Liu 2015; Zhang et al. 2017a). Although pandas have a simple digestive tract with no enzymes to digest the cellulose that is found in fibrous bamboo culms (Hu et al. 2010; Li et al. 2010; Wei et al. 2012; Nie et al. 2015), they do have adaptations that allow them to subsist on a bamboo diet such as enlarged molars and specialized gut microbes to aid in cellulose digestion (Zhu et al. 2011).

Pandas have also been shown to respond to subtle spatiotemporal variation in bamboo quality and quantity via movement and seasonal foraging patterns (Hong et al. 2016; Li et al. 2017). Although they feed on bamboo culms and leaves year-round, their diet is comprised almost completely of bamboo shoots (first-year bamboo) during shooting season in spring (Schaller et al. 1985; Dierenfeld 1997; Wei et al. 2000). Compared to culms and leaves, bamboo shoots have higher concentrations of nutrients and lower fiber content (Christian et al. 2015), with higher bioavailability of proteins, fats, minerals, and sugars for bamboo-specialized consumers (Schaller et al. 1985). The plant cell walls have not yet fully developed; therefore, shoots are more easily digested, and nutrients may be more bioavailable for giant pandas after foraging (Wei et al. 2000; Halvorson et al. 2010). As the height and age of a shoot increase, however, the lower part of the shoot begins to become hard and woody, and the shoot stumps left behind by giant pandas increase in height (Schaller et al. 1985).

Despite the wealth of existing knowledge on panda foraging, there has not yet been a study on the adaptation of giant pandas to spatiotemporal variation of bamboo shoots. To fill this knowledge gap, in this study, we examined giant panda foraging and movement patterns in relation to the spatiotemporal variation of umbrella bamboo (*Fargesia robusta*) growth across space and time in Wolong National Nature Reserve, Sichuan Province, southwest China. This study generated new information on the ecology of this threatened species, specifically by providing context for understanding how pandas relate to their dynamic environments.

## Materials and methods

### Study area

**Fig. 1:**
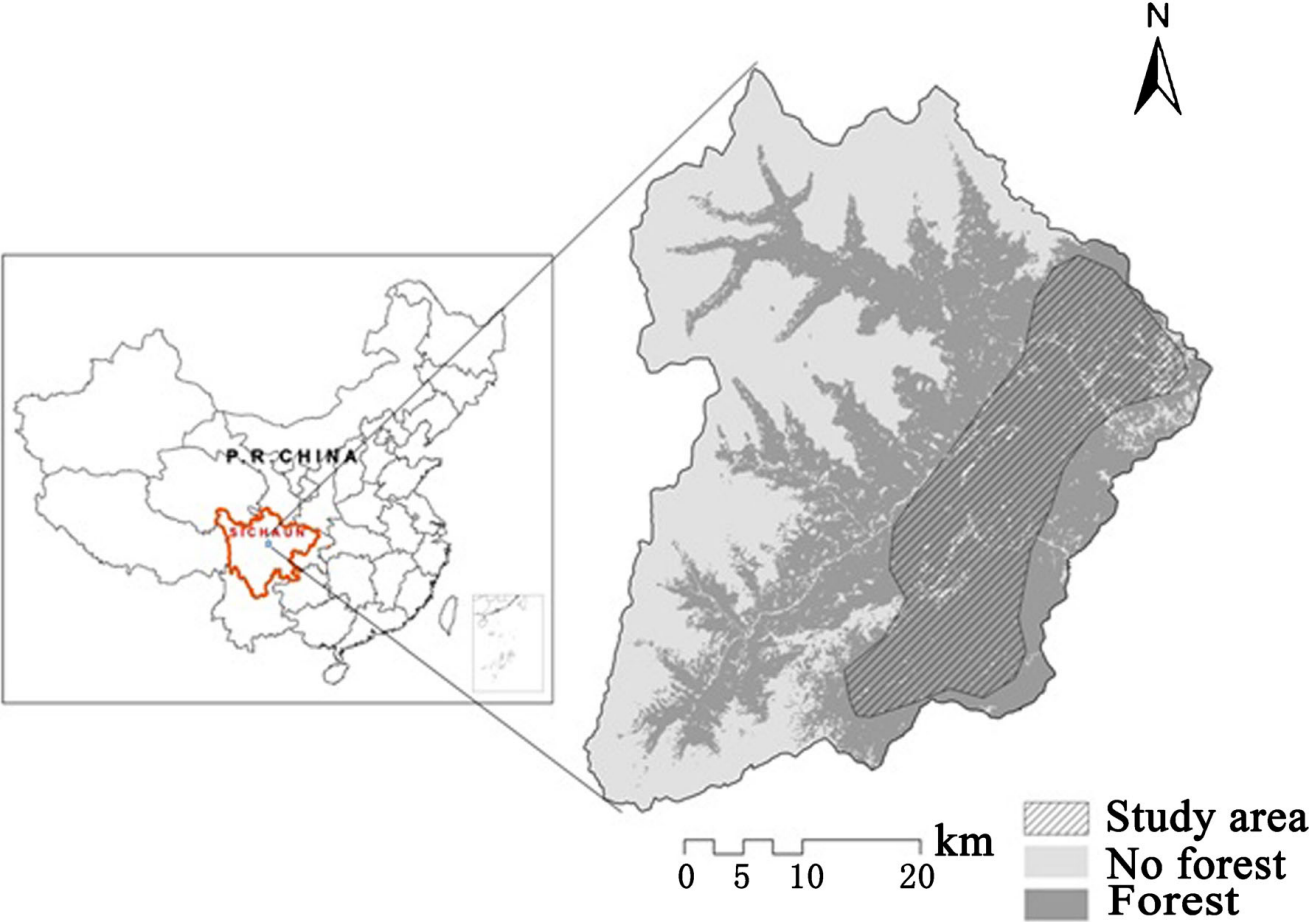
Study area in Wolong National Nature Reserve, China

The study was conducted in Wolong National Nature Reserve (102° 52′–103° 24′ E, 30° 45′–31° 25′ N), Sichuan Province, southwest China (Fig. 1). Wolong is located along the east margin of the Qinghai-Tibet Plateau and is one of the top 25 global biodiversity hot spots. The reserve was established in 1963 and is one of the earliest protected areas for giant pandas. This reserve covers about 2000 km^2^ of rugged ridges and narrow valleys spanning elevations ranging from 1200 to 6250 m.

The habitat for giant pandas consists of mixed coniferous and deciduous broadleaved forests and sub-alpine coniferous forests (Schaller et al. 1985). Two bamboo species, arrow bamboo (*Bashania faberi*) and umbrella bamboo (*F. robusta*), are dominant food sources of giant pandas in the reserve (Schaller et al. 1985). *B. faberi* mainly occurs above 2600 m in elevation (up to 3500 m) and *F. robusta* mainly at elevations of 1700 to 2600 m. Wolong was estimated to support over 100 wild giant pandas in the reserve in the fourth national survey (Sichuan Provincial Forestry Department 2015). Many other endangered and threatened animal species are living in this reserve, including the snub-nosed monkey (*Rhinopithecus roxellanae*), takin (*Budorcas taxicolor*), red panda (*Ailurus fulgens*), and red deer (*Cervus unicolor*).

### Data collection

In our study area, the diet of pandas was mainly composed of *F. robusta* shoots and the proportion was up to 93.5% during the spring season from April to June (Qin et al. 1993). Therefore, we sampled *F. robusta* bamboo shoots in 2015 during the spring shooting season (April to June). This is the time period over which shoots emerge and grow to near maximum height, after which point they harden and become less palatable as a food source for pandas. We established 16 transect lines following Hong et al. (2015), oriented from the valley to the ridge throughout our roughly 40-km^2^ study area in the northeast region of the reserve. Sampling plots (20 × 20 m^2^) were established along both sides of the transect lines no less than 100 m apart in elevation and not less than 200 m apart in horizontal distance. Five bamboo sub-plots (1 × 1 m^2^) were established in each plot (one in the center and one in each of the four corners). All bamboo sub-plots were marked with plastic wires to allow for resampling every 5 days from April to June. We subsequently measured the height and basal diameter of the bamboo shoots in each bamboo sub-plot. We also measured the height and basal diameter of foraged bamboo (both remaining shoot stumps and discarded shoot parts) at foraging sites encountered along the transect lines. We considered a foraging site to be a location where panda feces was deposited next to foraged bamboo within 2 weeks prior to the sampling time (determined by color and consistency of feces; Zhang et al. 2004; Hong et al. 2016). Simultaneously, dates and elevations of each bamboo sub-plots were also recorded.

To understand the movement patterns at a finer spatiotemporal scale, we collected elevation data from four giant pandas (Table 1) using GPS collars which recorded the animals’ locations every 4 h during April 2010 to June 2012 (Hull et al. 2015, 2016; Zhang et al. 2015, 2017b).

**Table 1:** Summary of GPS-collared pandas (*Ailuropoda melanoleuca*) in Wolong Nature Reserve, China

Panda	Age	Sex	Deployment date	Duration of tracking	Tracking months
Zhongzhong	Adult	F	10 Mar 2011	15 Mar 2011–30 Jul 2012	17
Meimei	Adult	F	29 Mar 2010	4 Apr 2010–20 Sep2011	18
Longlong	Sub-adult	F	04 Apr 2010	10 May 2010–10 Dec 2010; 10 Apr 2011–11 Oct 2011	15
Chuanchuan	Adult	M	31 Mar 2011	6 Apr 2011–27 Mar 2012	12

### Statistical analyses

To explore the growth patterns of *F. robusta* shoots over time and space, we plotted both bamboo height and basal diameter across both time and elevation and subsequently conducted curve estimation (optimum function estimator) to fit these relationships. Correlation coefficients were calculated via Pearson correlation analysis when data were normally distributed or Spearman correlation analysis when data were not normally distributed.

To uncover the availability of *F. robusta* shoots for giant pandas, firstly, the time points when the shoots reached 10 cm (T1), 30 cm (T2), and 200 cm (T3) were recorded. We chose these time points to represent germination (T1), initial availability of bamboo for panda foraging (T2), and last availability of bamboo for foraging (T3) based on previous studies (Schaller et al. 1985; Qin et al. 1993; Zhang et al. 2016). These time points were calculated through curve estimation of *F. robusta* shoot height over time (days). Area from the curve at T2 to that at T3 was defined as available time of *F. robusta* shoots for foraging by giant pandas. Finally, correlation between the time points and elevation was calculated through Spearman correlation analysis, and the analysis of impact of elevation on time was conducted by one-way ANOVA. All statistical tests were two-tailed and conducted in SPSS 17.0 (SPSS Inc. Chicago, USA), and the significance level of all analyses was 0.05.

We graphed the elevational migration pattern of the four GPS-collared giant pandas over time using GraphPad Prism 5 (GraphPad Software, Inc.). The shooting season was defined as the whole shoot phase of *F. robusta* shoots from initial shoot emergence in early April to the end of June. We only present the shooting season of 2011 because of available data on each giant panda.

## Results

### Bamboo shoot growth patterns

**Fig. 2:**
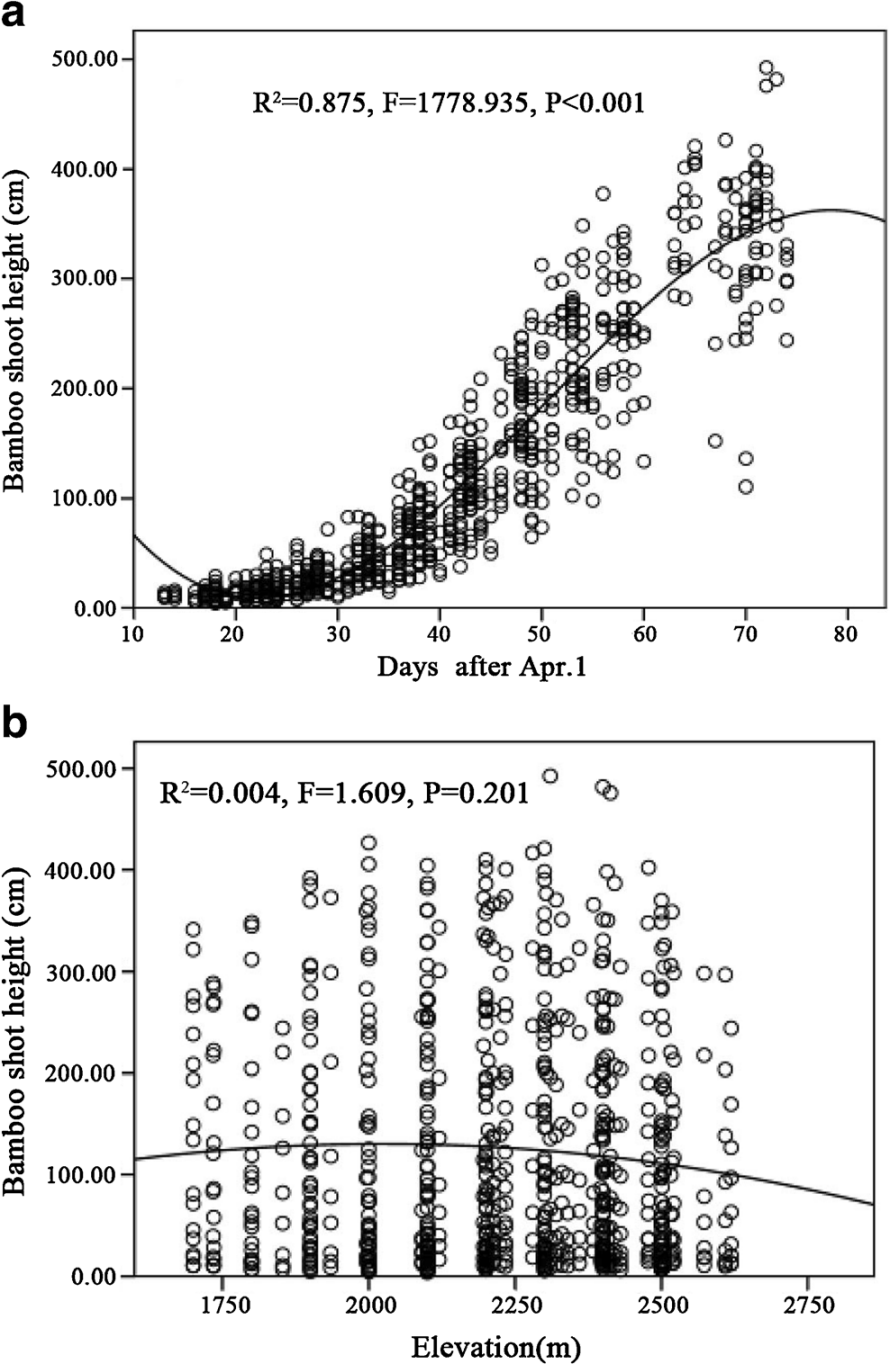
Fitted curves of the height of *F. robusta* bamboo shoots across time (**a**) and elevation (**b**)

**Fig. 3:**
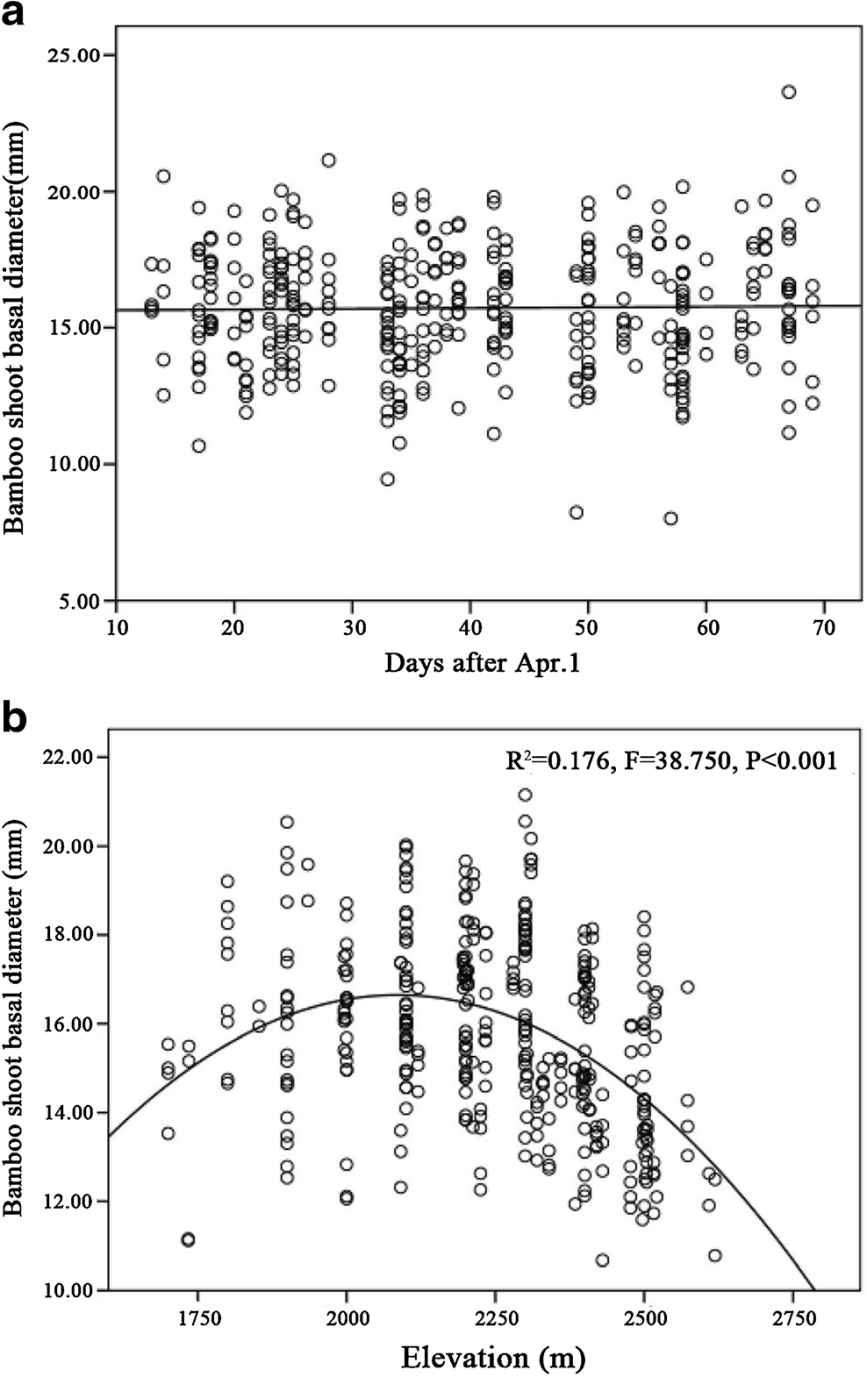
Fitted curves of the basal diameter of *F. robusta* bamboo shoots over time (**a**) and elevation (**b**)

The height of *F. robusta* shoots slowly increased in April and rapidly increased during May, after which growth then slowed again until the beginning of June (Fig. 2a; cubic function, *R*^2^ = 0.875, *P* < 0.001). There was no significant relationship between bamboo height and elevation (Fig. 2b) or bamboo diameter and time (Fig. 3a), but there was a significant quadratic relationship between elevation and basal diameter, with maximum diameter found at mid-elevation (Fig. 3b; *R*^2^ = 0.176, *P* < 0.001).

### Foraging patterns of giant pandas

**Fig. 4:**
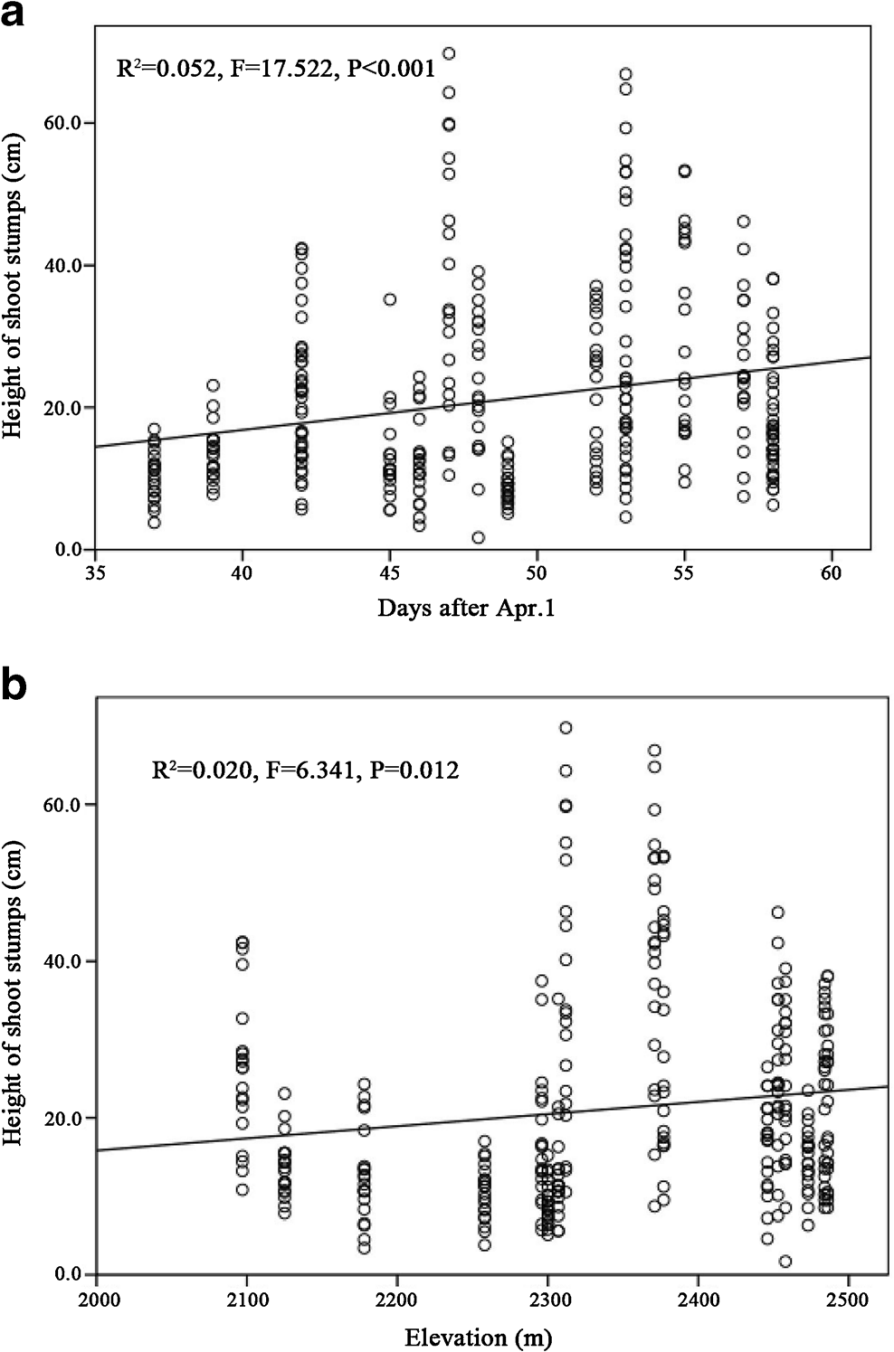
Fitted curves of the height of *F. robusta* bamboo shoot stumps left behind after panda foraging across time (**a**) and elevation (**b**)

**Fig. 5:**
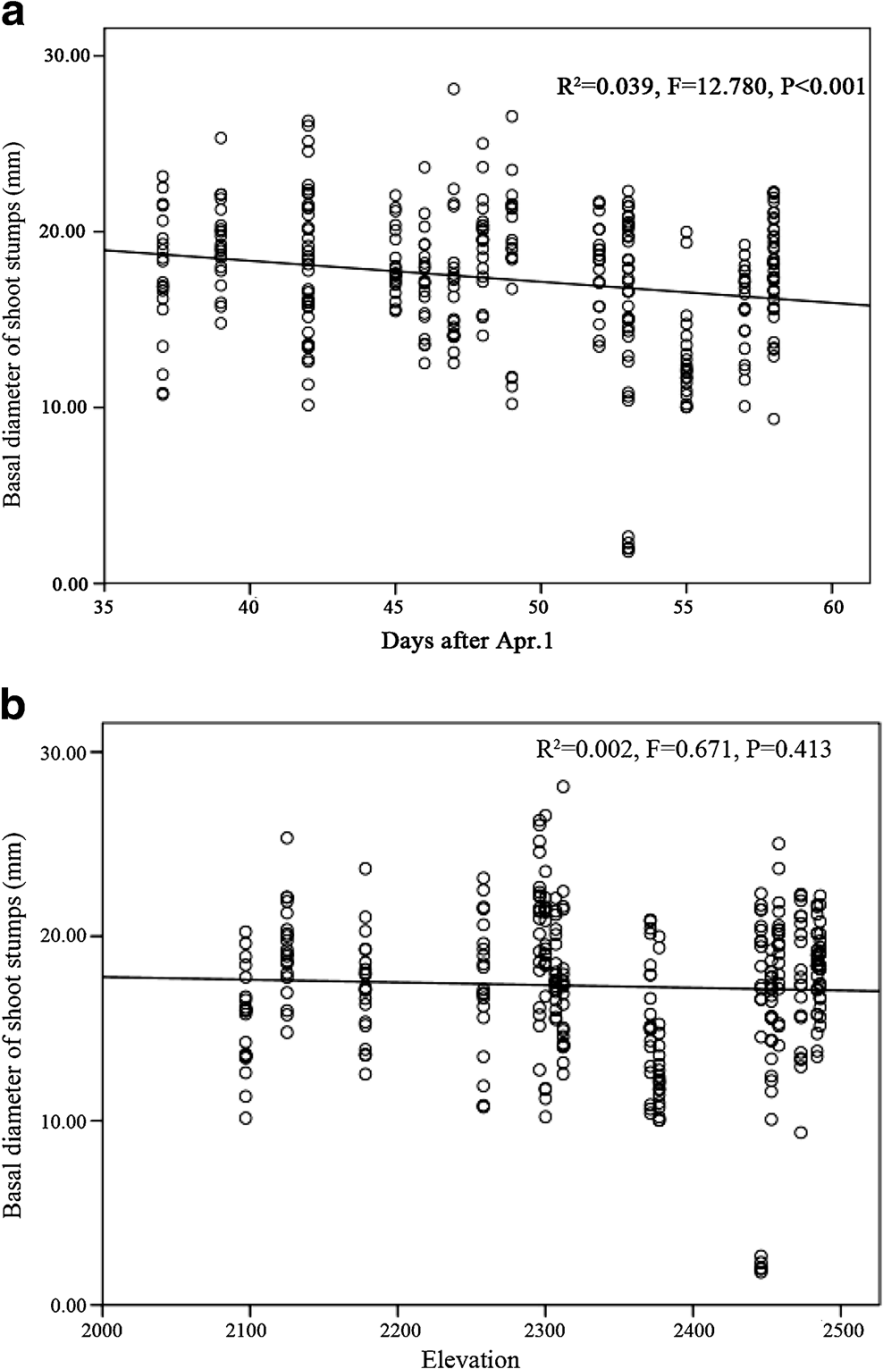
Fitted curves of the basal diameter of *F. robusta* bamboo shoot stumps left behind after panda foraging over time (**a**) and elevation (**b**)

**Fig. 6:**
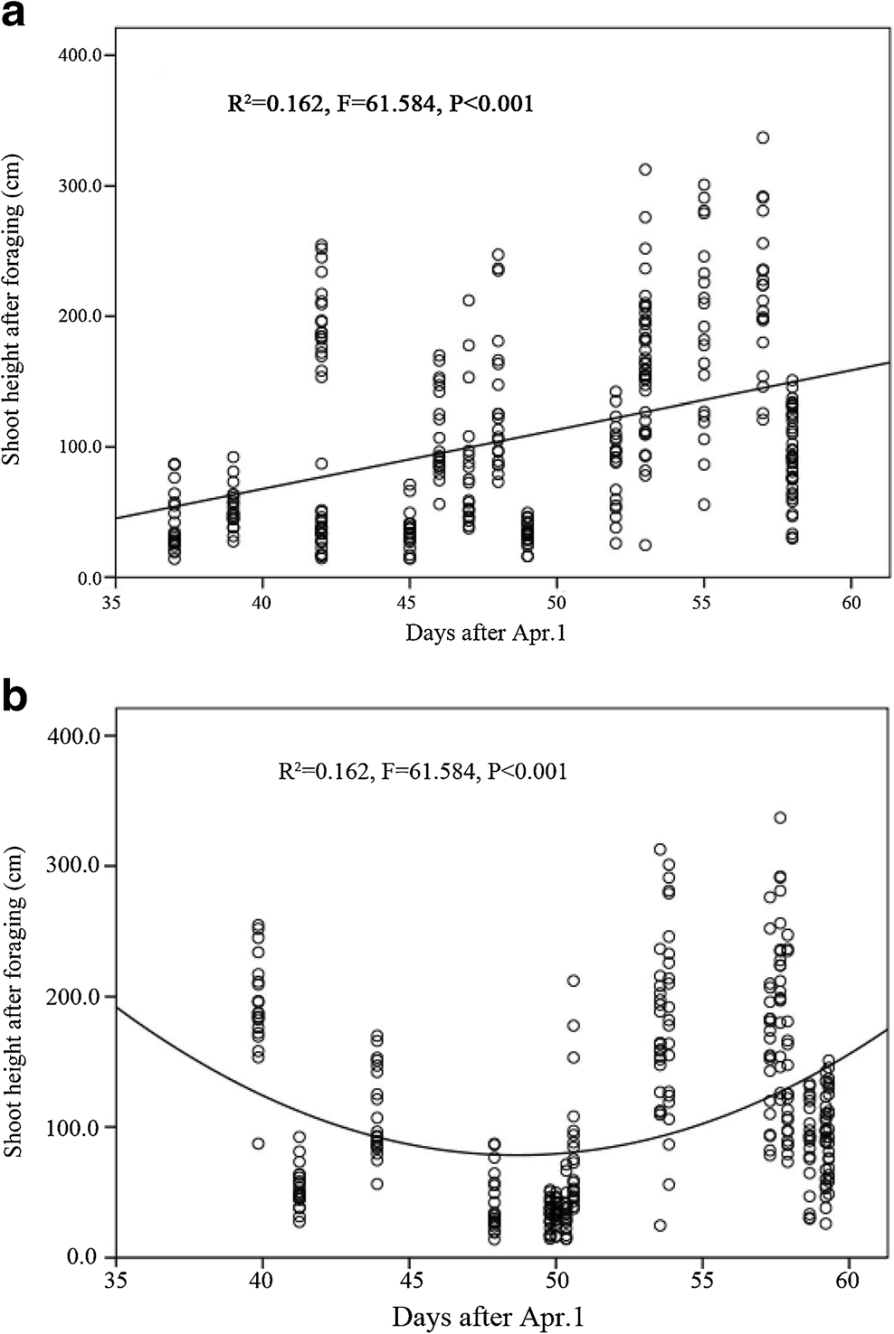
Fitted curves of the height of *F. robusta* bamboo shoots discarded by giant pandas over time (**a**) and elevation (**b**)

**Fig. 7:**
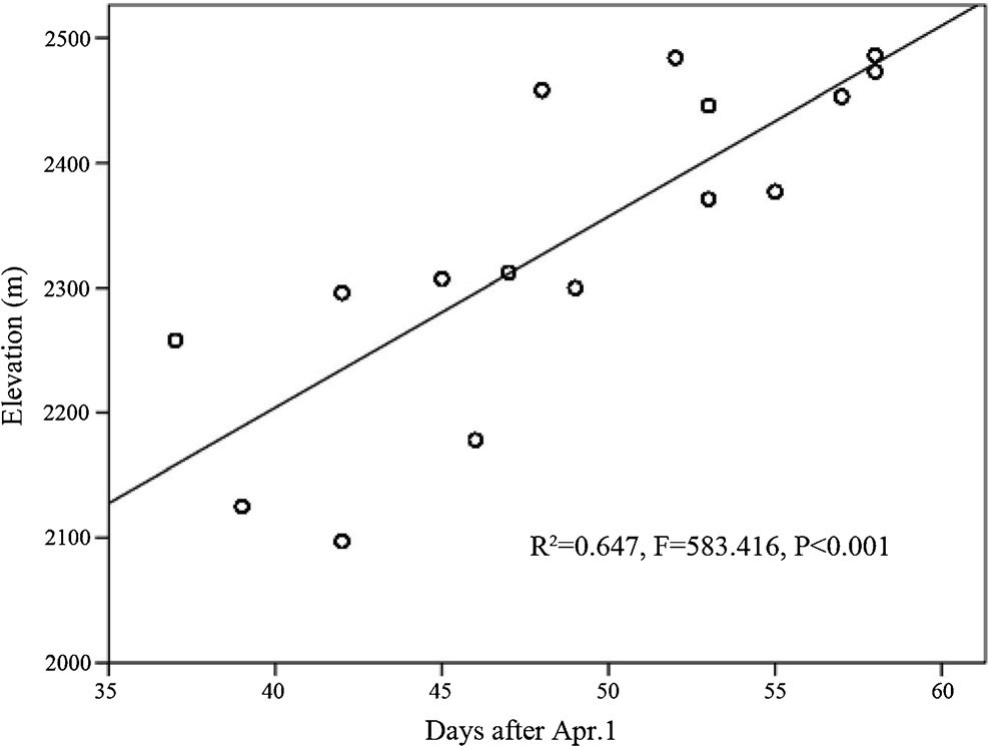
Linear estimation of the relationship between the time since the start of the growing season and the elevation of *F. robusta* bamboo shoot feeding sites used by giant pandas

The height of the shoot stumps remaining after being foraged by giant pandas was significantly higher as the shooting season progressed (Fig. 4a; *R* = 0.229, *P* < 0.001) and was positively related to elevation (Fig. 4b; *R* = 0.140, *P* = 0.012). Basal diameter of the foraged shoot stumps was significantly lower as the growing season progressed (Fig. 5a; *R* = − 0.197, *P* < 0.001) but is not correlated with elevation (Fig. 5b). The height of the remaining *F. robusta* shoot discarded after giant panda foraging significantly increased as the growing season progressed (Fig. 6a; *R* = 0.403, *P* < 0.001) and was the shortest at mid-elevation (Fig. 6b). The distribution of foraging sites found along transects suggested that the distribution of giant pandas along the elevation was significantly related to bamboo shoot growth as the season progressed (Fig. 7, *R*^2^ = 0.647, *P* < 0.001).

### Availability of *F. robusta* shoots for giant pandas

**Fig. 8:**
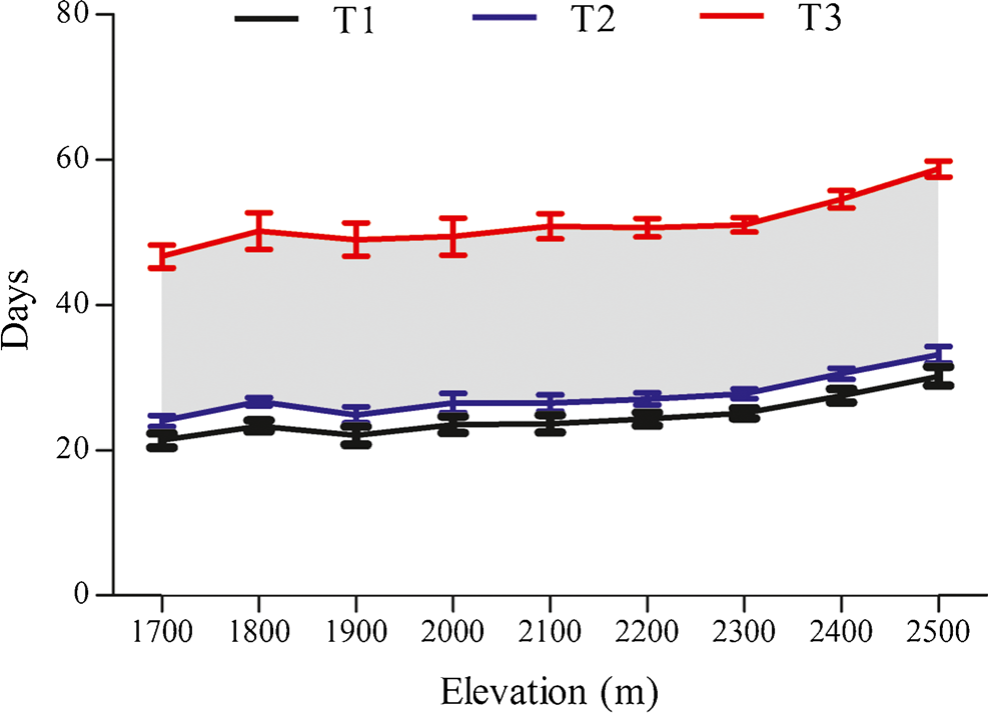
Availability of *F. robusta* shoots for giant pandas with increasing elevation. T1, T2 and T3 were the times at which *F. robusta* shoots reached 10, 30, and 200 cm in height, respectively (days since the start of the growing season). The shaded area from T2 to T3 was defined as the time during which *F. robusta* shoots were available for giant panda foraging

The timing of germination, initial availability of bamboo for foraging, and final availability of bamboo for foraging varied significantly across different elevations (T1: *df* = 8, *P* < 0.001; T2: *df* = 8, *P* < 0.001; T3: *df* = 8, *P* = 0.001) (Fig. 8). As elevation increased, the timing of each period shifted later (Fig. 8; *P* < 0.001), as did the total time for availability of bamboo shoots for pandas (T3–T2, gray area in Fig. 8).

### Giant panda elevational migration patterns

**Fig. 9:**
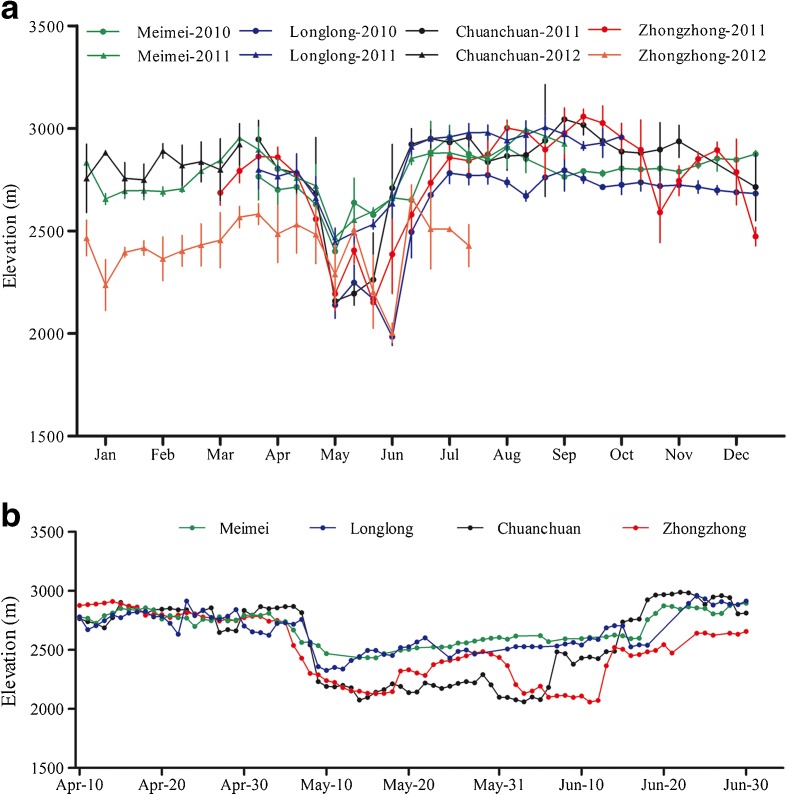
Elevational movement patterns of GPS-collared giant pandas across the year (**a**) and during the *F. robusta* bamboo shooting season (April–June) (**b**)

All four GPS-collared giant pandas began to shift down to the lower elevations where *F. robusta* grows in early April, reaching the lowest elevation points during the 2 months of May and June (Fig. 9a) and moving up again to above the distribution range of *B. fangiana* starting in mid-June (Fig. 9b). However, from early May to mid-June, there was variation across individuals, with two individuals showing more unpredictable movement patterns along this general pattern (Fig. 9a).

## Discussions

The growth of bamboo shoots is crucial for giant pandas during the shooting season in spring, when shoots make up almost all of their diet (Dierenfeld 1997; Christian et al. 2015; Liu 2015). Lower fiber content and higher concentrations of proteins, fats, minerals, and sugars of bamboo shoots are beneficial to bamboo-specialized consumers (Christian et al. 2015; Liu 2015). The shoots are more easily digested, and nutrients may be more bioavailable for giant pandas (Wei et al. 2000; Halvorson et al. 2010). During this season, higher 6-methoxy-2-benzoxazolinone (6-MBOA) content in bamboo shoots eaten by giant pandas contributes to augmenting immune defenses and may increase birth and survival rates (Shelby and Rosenfeld 2004). Advantageous compounds and high-proportion available energy in shoots may be the major impetus for migrational movements of giant pandas in this season, which also may account for the extensive energy intake strategy to procure them (Nie et al. 2015; Li et al. 2017).

Our findings on the cubic growth curve for *F. robusta* over the course of the shooting season are in line with previous studies (Schaller et al., 1985), as is the lack of significance of elevation in affecting bamboo height but with the largest basal diameters being found at mid-elevations (Schaller et al. 1985; Reid et al. 1991).

The new contributions of this study arose from tracking the changes in bamboo shoot growth and panda foraging over the course of both time and space. The findings show the dynamics in panda-bamboo space-time relationships that have not been previously articulated. For instance, the fact that we found a higher bamboo stump height of foraged bamboo with increasing elevation is likely related to the fact that pandas were foraging on higher elevations later in the season, when bamboo shoots become more fibrous and woody (Schaller et al. 1985). One other interesting finding was that the height of discarded shoots after giant panda foraging was the shortest at mid-elevation, suggesting a greater proportion of each shoot consumed at the elevation which supports the largest-diameter shoots (widely reported as panda’s preferred shoot characteristic, Schaller et al. 1985). Nonetheless, the basal diameter of stumps from foraged shoots was not significantly related to elevation and declined over time. This suggests that the well-documented panda use of larger-diameter shoots may not be as constant, as it is has sometimes been portrayed in the past, and instead varies over the course of the shooting season. Another nuanced observation was the fact that pandas moved down to the lowest part of the *F. robusta* range and later gradually made their way back up the elevation gradient to match the increasingly delayed window of availability of shoots as elevation increased (Schaller et al. 1985; Qin et al. 1993). In fact, the available time for foraging on *F. robusta* shoots was 1.51 days later for initial and 1.40 days later for final availability of foraging per 100 m in elevation (Fig. 8).

The elevational migration patterns that we documented suggest that pandas respond to forage quantity and quality of *F. robusta* shoots. This pattern of elevational migration has been previously documented at coarser scales via forest sampling and radio telemetry (Schaller et al. 1985) but not with the high spatiotemporal resolution of GPS collars (but see Zhang et al. 2015 for analysis of elevational migration in response to a different bamboo species in another part of giant panda habitat). The use of GPS collars allowed for more accurate comparisons across individual pandas, which showed marked individual variation in the migrational pathways. These differences could be due to the mating season in spring (Nie et al. 2012) and increasing home range and movement during this period for finding mates (Schaller et al. 1985; Zhang et al. 2014).

Our results highlight the importance of conserving *F. robusta* shoots in the reserve during spring seasons. *F. robusta* shoots are also a food source for local communities, and more research is needed in the future to determine the magnitude and impact of bamboo shoot collection activities on pandas. The shift of giant pandas from the high to the low elevation in early spring to forage on *F. robusta* suggests that protected area managers should take measures to lessen anthropogenic interference in this elevational range at this time of the year.

## References

[CR1] Christian AL, Knott KK, Vance CK, Falcone JF, Bauer LL, Fahey GC, Willard S, Kouba AJ (2015). Nutrient and mineral composition during shoot growth in seven species of Phyllostachys and Pseudosasa bamboo consumed by giant panda. J Anim Physiol An N.

[CR2] Clavel J, Julliard R, Devictor V (2011). Worldwide decline of specialist species: toward a global functional homogenization?. Front Ecol Environ.

[CR3] Dierenfeld ES (1997). Chemical composition of bamboo in relation to giant panda nutrition Linnean Society Symposium Series.

[CR4] Ducatez S, Clavel J, Lefebvre L (2015). Ecological generalism and behavioural innovation in birds: technical intelligence or the simple incorporation of new foods?. J Anim Ecol.

[CR5] Goss-Custard J, Caldow R, Clarke R, West A (1995). Deriving population parameters from individual variations in foraging behaviour. II. Model tests and population parameters. J Anim Ecol.

[CR6] Halvorson JJ, Cassida KA, Turner KE, Belesky DP (2010). Nutritive value of bamboo as browse for livestock. Renew Agr Food Syst.

[CR7] Hong MS, Yuan SB, Yang ZS, Yang XY, XD G, Huang F, Zhang ZJ (2015). Comparison of microhabitat selection and trace abundance of giant pandas between primary and secondary forests in Liziping Nature Reserve, China: effects of selective logging. Mamm Biol.

[CR8] Hong MS, Wei W, Yang ZS, Yuan SB, Yang XY, XD G, Huang F, Zhang ZJ (2016). Effects of timber harvesting on Arundinaria spanostachya bamboo and feeding-site selection by giant pandas in Liziping Nature Reserve, China. Forest Ecol Manag.

[CR9] Hu YB, Zhan XJ, Qi DW, Wei FW (2010). Spatial genetic structure and dispersal of giant pandas on a mountain-range scale. Conserv Genet.

[CR10] Hull V, Zhang JD, Zhou SQ, Huang JY, Viña A, Liu W, Tuanmu MN, Li RG, Liu D, WH X, Huang Y, Ouyang ZY, Zhang HM, Liu JG (2014). Impact of livestock on giant pandas and their habitat. J Nat Conserv.

[CR11] Hull V, Zhang JD, Zhou SQ, Huang JY, Li RG, Liu D, WH X, Huang Y, Ouyang ZY, Zhang HM (2015). Space use by endangered giant pandas. J Mammal.

[CR12] Hull V, Zhang JD, Huang JY, Zhou SQ, Viña A, Shortridge A, Li RG, Liu D, WH X, Ouyang ZY, Liu JG (2016). Habitat use and selection by giant pandas. PLoS One.

[CR13] Li RQ, FanW TG, Zhu HM, He L, Cai J (2010). The sequence and de novo assembly of the giant panda genome. Nature.

[CR14] Li YX, Swaisgood RR, Wei W, Nie YG, Hu YB, Yang XY, Gu XD, Zhang ZJ (2017). Withered on the stem: is bamboo a seasonally limiting resource for giant pandas?. Environ Sci Pollut Res.

[CR15] Liu JG (2015). Promises and perils for the panda. Science.

[CR16] Nie YG, Swaisgood RR, Zhang ZJ, YB H, Ma YS, Wei FW (2012). Giant panda scent-marking strategies in the wild: role of season, sex and marking surface. Anim Behav.

[CR17] Nie YG, Speakman JR, Wu Q, Zhang CL, Hu YB, Xia MH, Yan L, Hambly C, Wang L, Wei W, Zhang JG, Wei FW (2015). Exceptionally low daily energy expenditure in the bamboo-eating giant panda. Science.

[CR18] Owen-Smith N, Fryxell J, Merrill E (2010). Foraging theory upscaled: the behavioural ecology of herbivore movement. Philos T R Soc B.

[CR19] Qin ZS, Alan T, Cai XS (1993). Bamboo in giant pandas’ habitat and forest dynamic succession in Wolong.

[CR20] Reid D, Taylor A, Hu JC, Qin ZS (1991) Environmental influences on bamboo *Bashania fangiana* growth and implications for giant panda. J Appl Ecol 28:855–868

[CR21] Schaller GBHJC, Pan WS, Zhu J (1985). The giant pandas of Wolong.

[CR22] Shelby NJ, Rosenfeld MJ (2004) Methods for augmenting immune defenses contemplating the administration of phenolic and indoleamine-like compounds for use in animals and humans. United States Patent Application Publication, United States. Pub. No.: US 2004/0209877 Al

[CR23] Sichuan Provincial Forestry Department (2015). Sichuan’s giant panda—the report of the fourth giant panda survey of Sichuan Province.

[CR24] Simpson SJ, Raubenheimer D (2012). The nature of nutrition: a unifying framework from animal adaptations to human obesity.

[CR25] Slayter RA, Hirst M, Sexton JP, Kleijn D (2013). Niche breadth predicts geographical range size: a general ecological pattern. Ecol Lett.

[CR26] State Forestry Administration of the People’s Republic of China (2015) The giant pandas of China: status quo. Major findings of the fourth national survey on giant panda. http://www.forestry.gov.cn/main/4462/content-743596.html

[CR27] Wei FW, Wang ZW, Feng ZJ, Li M, Zhou A (2000). Seasonal energy utilization in bamboo by the red panda (*Ailurus fulgens*). Zoo Biol.

[CR28] Wei FW, YB H, Zhu LF, Bruford MW, Zhan XJ, Zhang L (2012). Black and white and read all over: the past, present and future of giant panda genetics. Mol Ecol.

[CR29] Zhang ZJ, Wei FW, Li M, Zhang BW, Liu XH, Hu JC (2004). Microhabitat separation during winter among sympatric giant pandas, red pandas, and tufted deer: the effects of diet, body size, and energy metabolism. Can J Zool.

[CR30] Zhang ZJ, Sheppard JK, Swaisgood RR (2014). Ecological scale and seasonal heterogeneity in the spatial behaviors of giant pandas. Integr Zool.

[CR31] Zhang JD, Hull V, Huang JY, Zhou SQ, WH X, Yang HB, William JM, Li RG, Liu D, Huang Y, Ouyang ZY, Zhang HM, Liu JG (2015). Activity patterns of the giant panda (Ailuropoda melanoleuca). J Mammal.

[CR32] Zhang MC, Huang JY, Huang Y, Li DS, Liu D, Zhou XP, Xie H, He SS, Zhou YM, Zhang HM (2016). Variations of the Fargesia robusta shoots at different elevations in Wolong Nature Reserve, China. J China West Normal Univ (Nat Sci).

[CR33] Zhang JD, Hull V, Ouyang ZY, Li RG, Connor T, Yang HB, Zhang ZJ, Silet B, Zhang HM, Liu JG (2017). Divergent responses of sympatric species to livestock encroachment at fine spatiotemporal scales. Biol Conserv.

[CR34] Zhang JD, Vull V, Ouyang ZY, He L, Connor T, Yang HB, Huang JY, Zhou SQ, Zhang ZJ, Zhou CQ, Zhang HM, Liu JG (2017b) Modeling activity patterns of wildlife using time-series analysis. Eco Evol 7(8):2575–2584 10.1002/ece3.2873 PMC5395454 28428848

[CR35] Zhu LF, Wu Q, Dai JY, Zhang SN, Wei FW (2011). Evidence of cellulose metabolism by the giant panda gut microbiome. PNAS.

